# Long-term quality of life outcomes following robotic inguinal hernioplasty: a single-centre experience of 100 cases

**DOI:** 10.1007/s11701-025-02317-1

**Published:** 2025-04-15

**Authors:** Krishna Kotecha, Chun Khai Loh, Nazim Bhimani, Alex Boue, Jaswinder S. Samra, Anubhav Mittal

**Affiliations:** 1 Department of Upper Gastrointestinal Surgery, Royal North Shore Hospital, St Leonards, NSW 2065 Australia; 2 Northern Clinical School, University of Sydney, Sydney, 2006 Australia; 3 Faculty of Medicine and Health, University of Sydney, Sydney, NSW Australia; 4 School of Medicine, University of Notre Dame, Sydney, Australia

**Keywords:** Inguinal hernia, Robotic surgery, Quality of life

## Abstract

To evaluate the long-term efficacy and health-related quality-of-life (HRQoL) outcomes in patients who underwent robotic-assisted transabdominal preperitoneal (R-TAPP) inguinal hernioplasty in an Australian setting. A cross-sectional analysis of patients who underwent R-TAPP inguinal hernioplasty was performed. Health-related quality of life was assessed using the SF-36 questionnaire. Post-operative outcomes, including chronic pain, opioid requirements, hernia recurrence, and return to activities, were evaluated. A total of 100 patients who completed the SF-36 questionnaire (94 males, 6 females) were included in the study. The median age at operation was 64 years. Of the 77 patients who answered the question on opioid use, 56% used opioids (8% for less than 1 week, 19% for 1 week, 17% for between 1 and 2 weeks, and 9% for more than 2 weeks). Eighty-five percent reported no chronic groin pain at follow-up. The hernia recurrence rate was 1%. The median follow-up period was 35 months. The median time to return to work, driving, and exercise was 1, 2, and 4 weeks, respectively. SF-36 scores showed optimal outcomes (median 100) in physical functioning, role limitations, and social functioning domains. R-TAPP inguinal hernioplasty demonstrates excellent long-term outcomes with high health-related quality-of-life (HRQoL) scores, low chronic pain rates, and minimal recurrence rates. There is the potential for increasing uptake of this procedure with increasing availability and decreasing cost of robotic surgery technology.

## Introduction

Inguinal hernia accounts for 75% of all abdominal hernias, with an estimated lifetime risk of 27% and 3% in males and females, respectively [1], peaking after the age of 70 in adults [2]. Whilst the Lichtenstein tension-free repair has long been considered the gold standard [3], the surgical approach to inguinal hernia repair has evolved significantly over recent decades.

Open repair of inguinal hernia is associated with increased post-operative pain and longer recovery periods compared to minimally invasive approaches [4]. The advent of laparoscopic techniques, particularly transabdominal preperitoneal (TAPP) and total extraperitoneal (TEP) repairs, has demonstrated superior outcomes in terms of post-operative pain, surgical site infections, and return to function [5]. Robotic-assisted platforms offer the surgeon enhanced three-dimensional visualisation and improved instrument articulation with a comparable safety profile to laparoscopic repair [6]. Although post-operative outcome measures compare similarly to laparoscopic surgery, endorsement of robotic-assisted inguinal hernia repair by surgeons and policymakers has not been unanimous, particularly when considering factors explored in the literature, such as cost-effectiveness, learning curve, and long-term outcomes [7]. However, patients have reported high satisfaction rates with robotic surgery of other organ systems [8, 9], and improved post-operative pain scores, physical and social functioning, and early return to normal activities.

Therefore, this study aims to evaluate the long-term efficacy and health-related quality-of-life (HRQoL) outcomes following robotic-assisted inguinal hernioplasty in the largest retrospective Australian study to date.

## Methods

### Study design

Following approval by an institutional ethics committee (2022/ETH02245), a retrospective analysis of prospectively collected data was conducted at a single tertiary referral centre between January 2018 and December 2023. Adult patients aged over 18 years who underwent robotic-assisted unilateral inguinal hernioplasty (recurrent and primary), and consented to completing the HRQoL assessment, were included in the study. Patients under the age of 18 years or those who underwent hernia repair without mesh placement or requiring conversion to an open procedure were excluded from this study.

### Data collection

Demographic data, clinical characteristics, and perioperative outcomes were prospectively recorded in a standardised database. HRQoL was assessed using the validated SF-36 questionnaire (version 1.0) [10] through telephone follow-up between 2023 and 2024. The SF-36 questionnaire data collection includes;

- Chronic pain persistence and intensity (as defined by the SF-36)
- Opioid analgesia requirements
- Hernia recurrence
- Time to return to normal activities.

### Outcome measures

- Long-term HRQoL
- Chronic post-operative pain
- Hernia recurrence rate
- Post-operative complications
- Return to normal activities.

### Surgical technique

All hernia defects were reinforced with a ProGrip laparoscopic self-fixating mesh (Medtronic, Minneapolis MN). The robotic platform used was the da Vinci XI device (Intuitive Medical, Sunnyvale CA), and all repairs were performed by a single, high-volume hepatopancreatobiliary (HPB) surgeon, with extensive experience using and instructing the use of the robotic platform. A supraumbilical port and right and left upper abdomen ports were inserted. A preperitoneal plane was developed from the medial umbilical ligament extending laterally to the area of defect, with further dissection in the Bogros and Retzius spaces to facilitate sac reduction and delineation of hernia anatomy. The mesh was then appropriately contoured, cut to size (generally 11 × 9 cm), and positioned. The peritoneal flap was closed with barbed absorbable 3–0 V-loc suture.

### Statistical analysis

Continuous variables were expressed as medians with ranges for non-normally distributed data and mean and standard deviations for normally distributed data. Categorical variables were presented as counts and percentages. All statistical analyses were performed using Stata® BE for Windows® version 17.1 (StataCorp, College Station, Texas, USA).

## Results

A total of 100 patients underwent unilateral R-TAPP inguinal hernioplasties between 2018 and 2023, and successfully completed the SF-36 questionnaire. The patients included in the analysis consisted of 94 males (94%) and 6 females (6%) with a median age of 64 years (range 30–84) at the time of operation. Six (6%) patients had a radical prostatectomy and 18 (18%) had at least one intraabdominal surgery previously. A total of 19 (19%) suffered from chronic pain before their inguinal hernia repairs, as defined by the ICD-11. The median age at the completion of survey was 66 years old (range 34–86), with a median follow-up of 35 months (range 2–71).

Of the 19 patients who reported chronic pain, 5 reported pain unrelated to inguinal hernia (neck arthritis, chronic back pain from previous L5 laminectomy and T11/12 disc prolapse, non-specific low back pain, right mid upper back pain, and stress-related headache/neck pain). The patient who had chronic laminectomy related pain, required regular long-term non-opioid analgesia. Of the remaining 14 of 19 patients with chronic groin pain, only 2 had previous surgery. One patient had undergone a laparoscopic left nephrectomy (and required long-term non-opioid analgesia) and the other an open right inguinal hernia repair with radical prostatectomy (with no long-term analgesia required).

Of the 18 patients who had previous intra-abdominal surgery, the most common procedure was a laparoscopic appendicectomy (12 of 18 patients, 67%).

Of the 100 patients, only 77 answered questions about post-operative opioid usage. Of these 77, opioid analgesia was required in only 26 (34%) patients for varying durations post-operatively: 2 patients (8%) for less than 1 week; 15 patients (19%) for up to a week; and 9 patients (12%) for ≥ 2 weeks. A total of 85 patients (85%) reported no groin pain on follow-up. Of the remainder who had pain, 2 patients (2%) reported pain episodes daily; 5 (5%) patients weekly, 2 patients (2%) monthly; and 6 patients (6%) occasionally. The median pain score in those who experienced pain was 3 (range 2–8). The pain did not interfere with their daily living.

The median number of weeks required to return to work, driving and exercise was 1 (range 0–13), 2 (range 0–13), and 4 (range 0–36) respectively (Table 1). Of SF-36-measured HRQoL, a median score of 100 was achieved in the domains of physical functioning, role limitations due to physical health, role limitations due to emotional problems and social functioning; 90 in pain; 88 in emotional well-being; 75 in general health; and 70 in energy or fatigue (Table 2). One patient had a superficial wound infection requiring oral antibiotics (Clavien–Dindo 2).

**Table 1:** Descriptive statistics

Variable	N = 100 (%)
Gender, *n* (%) Male Female	94 (94%) 6 (6%)
Median age at operation (range)	64 (30–84)
Median age at completion of survey (range)	66 (34–86)
Median follow-up from operation in months (range)	35 (2–71)
Previous surgery Radical prostatectomy before inguinal hernia, *n* (%) Intra-abdominal surgery before inguinal hernia, *n* (%)	24 (24%) 6 (6%) 18 (18%)
Answered questions about opioid usage Opioid use frequency number (%) < 1 week 1 week 1–2 weeks ≥ 2 weeks	N = 77 N = 43 (56%) 2 (8%) 15 (19%) 17 (17%) 9 (12%)
Hernia recurrence, number (%) No Yes	99 (99%) 1 (1%)
Groin pain, Number (%) No Yes, on the same side Yes, on the other side Yes, on both sides	85 (85%) 11 (11%) 2 (2%) 2 (2%)
Frequency of pain (from above), number (%) Daily Weekly Monthly Occasional	2 (13%) 5 (34%) 2 (13%) 6 (40%)
Median pain score (range) in patients who experience pain Number (%)	3 (2–8%)
Pain medications on a regular basis, number (%)	1 (1%)
Chronic pain before your operation, number (%)	19 (19%)
How long in weeks did it take to return to driving? (range)	2 (0–13%)
How long in weeks did it take to return to exercise? (range)	4 (0–36%)
How long in weeks did it take to return to work? (range)	1 (0–13%)

**Table 2:** SF-36 results

	Median (range)	Mean (s.d.)
Physical functioning	100 (15–100)	92.0 (15.0)
Role limitations due to physical health	100 (0–100)	90.8 (25.3)
Role limitations due to emotional problems	100 (0–100)	94.0 (20.3)
Energy/fatigue	70 (15–100)	68.0 (18.3)
Emotional well-being	88 (36–100)	82.5 (14.7)
Social functioning	100 (37.5–100)	92.4 (14.0)
Pain	90 (32.5–100)	88.1 (15.7)
General health	75 (30–100)	74.1 (17.7)

## Discussion

The study examined the efficacy and HRQoL outcomes in patients who underwent robotic-assisted transabdominal preperitoneal (R-TAPP) inguinal hernioplasty at a single Australian institution, the largest Australian study to date. Using the SF-36 questionnaire, the study assessed HRQoL, chronic pain, opioid requirements, hernia recurrence, and return to activities in 100 patients with a median follow-up period of 35 months.

The open, tension-free inguinal hernia repair carries a risk of injury or mesh entrapment to the ilioinguinal, iliohypogastric, and genitofemoral nerves, with 9–15% [11] incidence of resultant chronic groin pain. Hajude et al. recently evaluated patient’s HRQoL following open mesh inguinal hernioplasty by means of SF-36 questionnaire and found significant improvement in all domains at 1- and 6-month follow-ups when compared to pre-operative baseline [12]. Similar findings are reported by Iftikhar et al., where satisfactory HRQoL (> 50 in all domains of SF-36) was achieved in 81% of those who had a unilateral open mesh inguinal hernia repair [13]. However, nearly 50% of patients had moderate-to-severe pain at 4 week follow-up versus 15% reported in our study, hence underscoring the likely disadvantage of an open, invasive approach to inguinal hernioplasty especially in the post-operative recovery course. A multi-centre propensity-matched study performed by Lima et al. [14] shows no difference between open, laparoscopic and robotic approaches in terms of readmission, reoperation, and surgical site infection at 30 days. However, the popularity and accessibility of the laparoscopic approaches (TEP and TAPP) to inguinal hernia repair are due to reduced post-operative pain, shorter recovery period, and the flexibility of detecting or repairing incidental contralateral hernia in the same operation [15–17].

However, the adoption of robotic technology allows superior visual clarity of operative field, increased dexterity with seamless manoeuvrability, and acute precision of wristed instrumentation to aid performance of minimally invasive yet complex operations [18, 19]. Kudsi et al. prospectively compared the outcomes of laparoscopic totally extraperitoneal repair versus robotic-assisted TAPP hernioplasties, and found the mean operation duration, and intra- and post-operative complications were largely identical between the two groups despite the significantly greater number of complex cases in the robotic-assisted TAPP arm [20]. However, the study was undertaken during the early period of transition from L-TEP to R-TAPP based on a single surgeon’s experience. Another case series of 97 R-TAPP patients by Morrell et al. reported zero conversion rate as well as hernia recurrence at a mean follow-up of approximately 23 months [21]. Peltrini et al.’s multi-centre study with propensity score matching analysis found comparable outcomes between R-TAPP and L-TAPP in terms of complication rates, post-operative pain and hernia recurrence, not mentioning shorter length of hospital stay in the former [22]. However, in keeping with findings from Waite et al., R-TAPP was frequently associated with a longer operative time, likely attributable to the learning curve inherent to the use of a novel robotic platform [22, 23].

A retrospective study by Gundogdu et al. [24] compared R-TAPP and LTEP approaches for bilateral inguinal hernia repair. The cohort included 49 patients (33 LTEP, 16 RTAPP) with similar demographics and comorbidities. The RTAPP cohort had significantly longer operative time, similar or lower post-operative pain scores with no recurrences in either group at follow-up, lower complication rate, similar length of stay, and higher costs. Similar findings were found in a 2021 [25] systematic review and meta-analysis that compared outcomes across 19 studies comparing 4248 open repairs, 2521 had robotic repairs and 1495 laparoscopic repairs. Most studies reported cases performed on Da Vinci systems, with few series reporting outcomes from the Dexter system (Distalmotion; Epalinges, Switzerland), and case reports/technical papers on the use of the Versius (CMR Surgical; Cambridge, United Kingdom), and Hugo (Medtronic; Minneapolis, Unites States).

Despite early promising data on R-TAPP, there exist concerns regarding its long-term impacts on important parameters, such as HRQoL, chronic pain, and hernia recurrence. The practical advantage of robotic surgery over the laparoscopic techniques in inguinal hernioplasty remains an area of keen research interest. Of note, the RIVAL trial by Prahbu et al. reported the lack of benefit in using the robotic approach to simple inguinal hernioplasties compared with the laparoscopic technique, given no significant differences in the rate of wound complications, re-hospitalisation, post-operative pain, and HRQoL pre-operatively, at 1-week and 1-month follow-ups. The robotic approach resulted in higher costs, not to mention increased surgeon frustration and negligible ergonomic advantage [26].

**Fig. 1:**
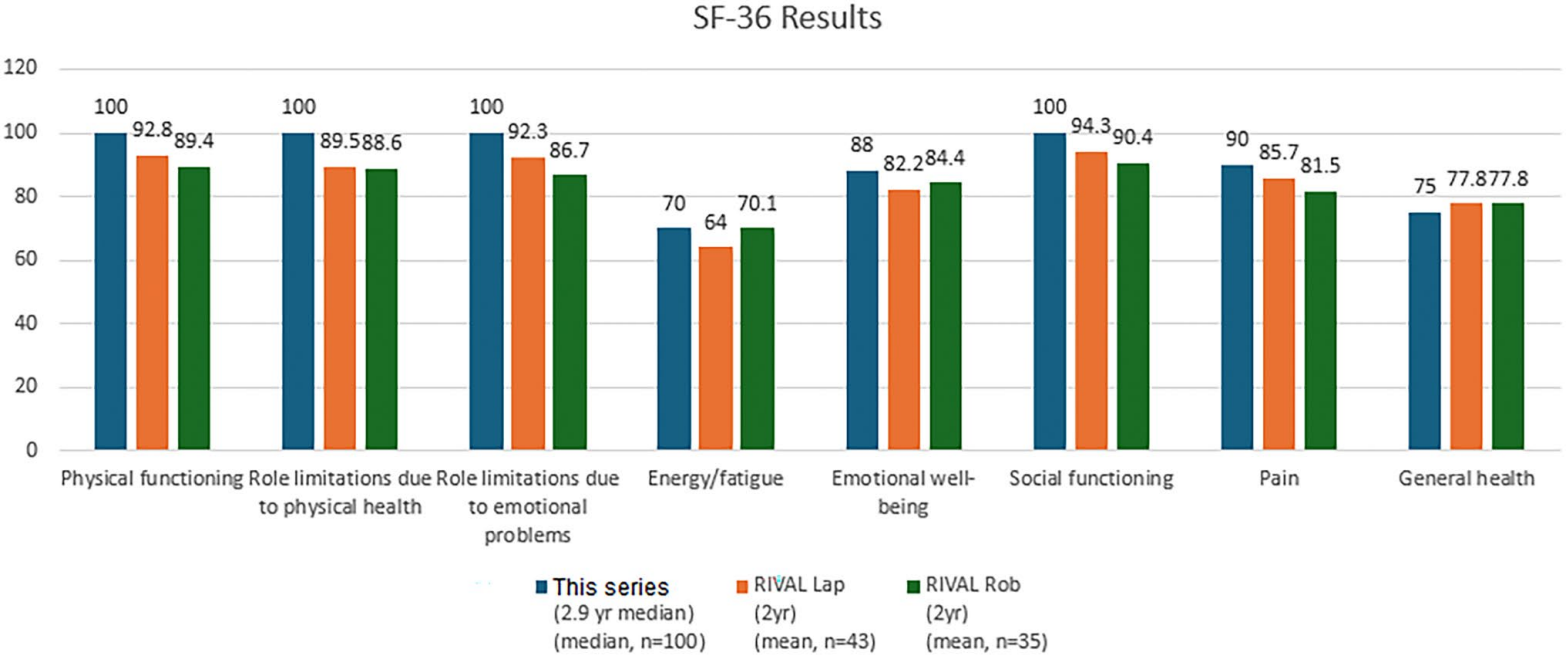
Comparison of QoL outcomes to RIVAL series

Our study, conducted in a more recent era with increased surgical robotic experience, yielded improved findings relating to most SF-36 domains of HRQoL. For reference, these results are displayed against those of the RIVAL trial laparoscopic and robotics cases at 1- and 2-year follow-ups (Fig. 1). Similarly, when analysed against the results obtained by Chuah et al. and Fedorkiv et al., our study findings on HRQoL following R-TAPP are similar to those of patients who underwent a laparoscopic repair, highlighting the long-term efficacy of such novel, minimally invasive approach [27, 28]. The extent to which the full benefits of robotic-assisted inguinal hernioplasty could be realised hinges upon crucial factors such as surgeon experience and patient selection strategies. Indeed, Awad et al. retrospectively reviewed the outcomes of R-TAPP performed by a larger cohort of surgeons (*n* = 10) at disparate stages of experience and found late experience (remainder of each surgeon’s experience following the first 20 cases) was associated with not only less operative time and fewer complications but also a lower direct operative cost per case [28]. Such findings mirrored that of our study, where all the cases were performed by a single high-volume upper gastrointestinal surgeon with extensive experience in the use of robotic-assisted platform.

## Limitations

This study is limited by a number of factors. A larger sample size could be used to reduce margins of error and standard of deviations for low-probability events, such as chronic pain and hernia recurrence. There was attrition in completing the full survey, particularly in answering questions about opioid use. Patients included in this study were from a single institution, which might in turn reduce the external validity of our findings. Critics of the robotic approach, and robotic surgery point to its increased cost compared to laparoscopic surgery. [29, 30] Clinical correlation, rather than telephone follow-up, would have been more useful in evaluating patients who self-report a hernia recurrence; due to the geography and population density of Australia, in-person clinical follow-up is not always possible. A multi-centre, randomised-controlled prospective trial with a larger subject cohort would prove invaluable in confidently determining the relative benefits of R-TAPP over laparoscopic techniques on long-term patient outcomes, as well as performing a detailed cost comparison.

## Conclusion

This is the largest Australian study demonstrating the long-term efficacy of R-TAPP inguinal hernioplasty with benefits on HRQoL. When performed by surgeons with appropriate experience in the robotic platforms, it is associated with a short recovery time and low rate of recurrence. Future research should focus on multi-centre, randomised-controlled trials comparing the robotic versus laparoscopic techniques with cost analysis in complex inguinal hernia cases to evaluate their relative benefits in hernioplasty.

## Data Availability

No datasets were generated or analysed during the current study.
